# Supplementary material for: The metazoan landscape of mitochondrial DNA gene order and content is shaped by selection and affects mitochondrial transcription
Source: Commun Biol. 2023 Jan 23;6:93. doi: 10.1038/s42003-023-04471-4 (PMC9871016; doi:10.1038/s42003-023-04471-4)
Supplement: Supplementary file 2 — Supplementary Information [file 42003_2023_4471_MOESM2_ESM.pdf]

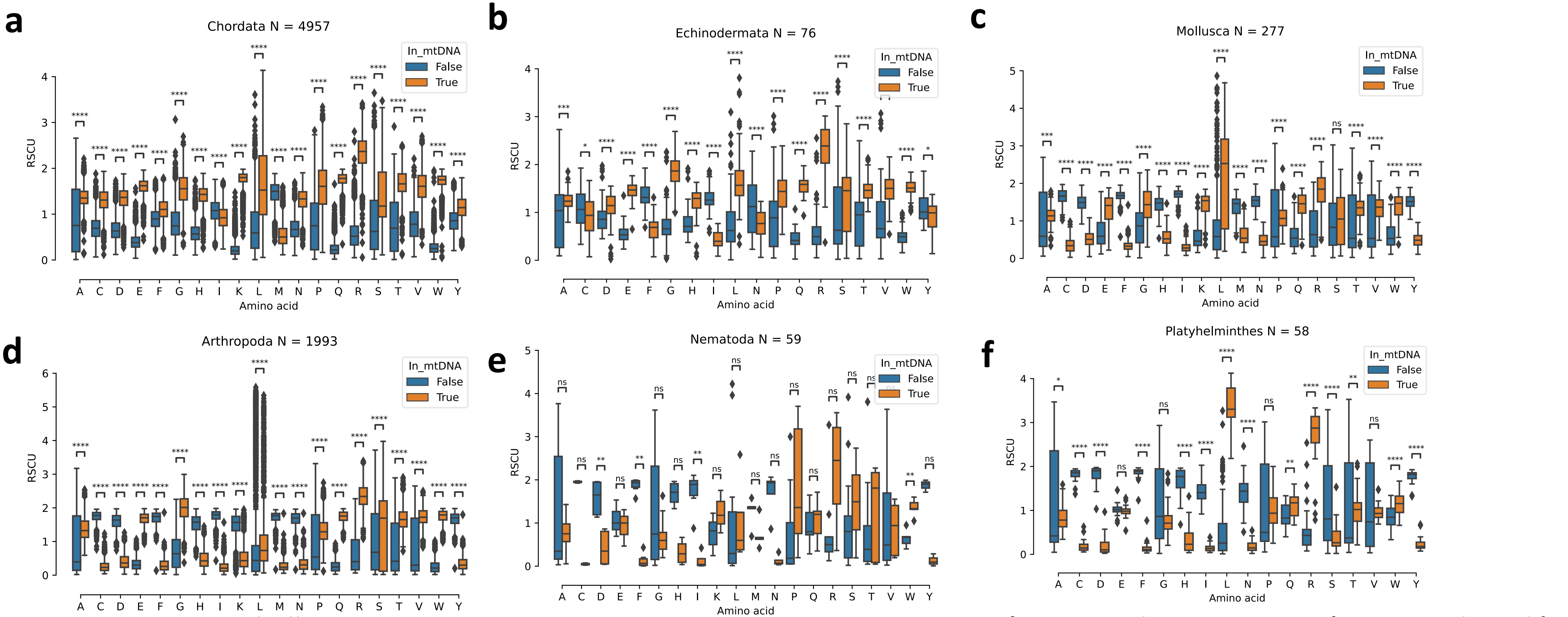

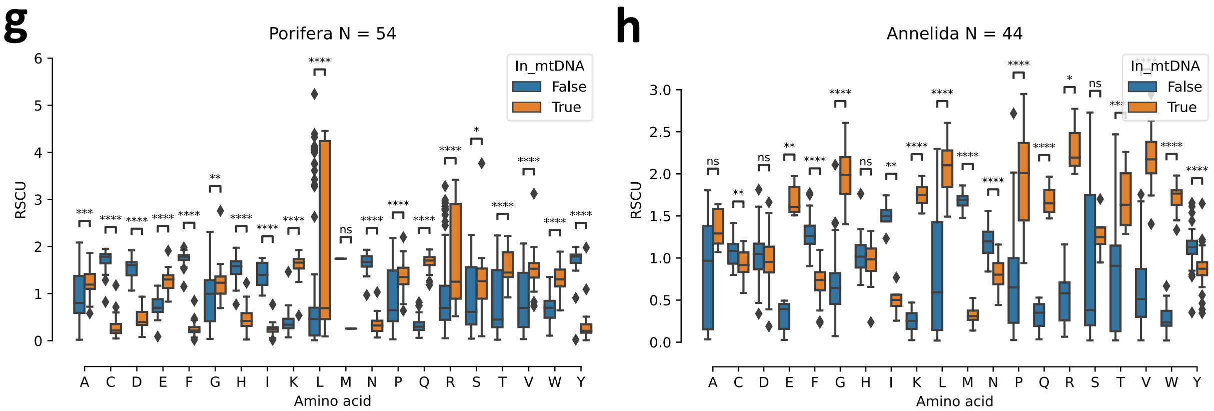

Supplementary Figure S1. RSCU values per-amino acid for major phyla.

Boxplot comparisons of RSCU values between codons recognized (orange) and unrecognized (blue) by the mt-tRNA, per amino acid, and for each studied phylum. The amino acids were filtered as follows: these having at least two different codons, one recognized and one unrecognized by the mt-tRNAs. Studied phyla should have at least two mt-tRNA genes which associate with amino acids that can be translated by at least two different codons. The RSCU values were compared using an FDR-corrected two-sided Mann-Whitney test. P-value ranges are indicated by asterisks. *: 0.01 < p <= 0.05, **: 0.001 <= p < 0.01, ***: 0.0001 < p <= 0.001, ****: p <= 0.0001 . Each box extends from the 25th to the 75th percentiles, whiskers denote values within 1.5 interquartile range of the percentiles. Dots denote outliers outside of mentioned ranges.

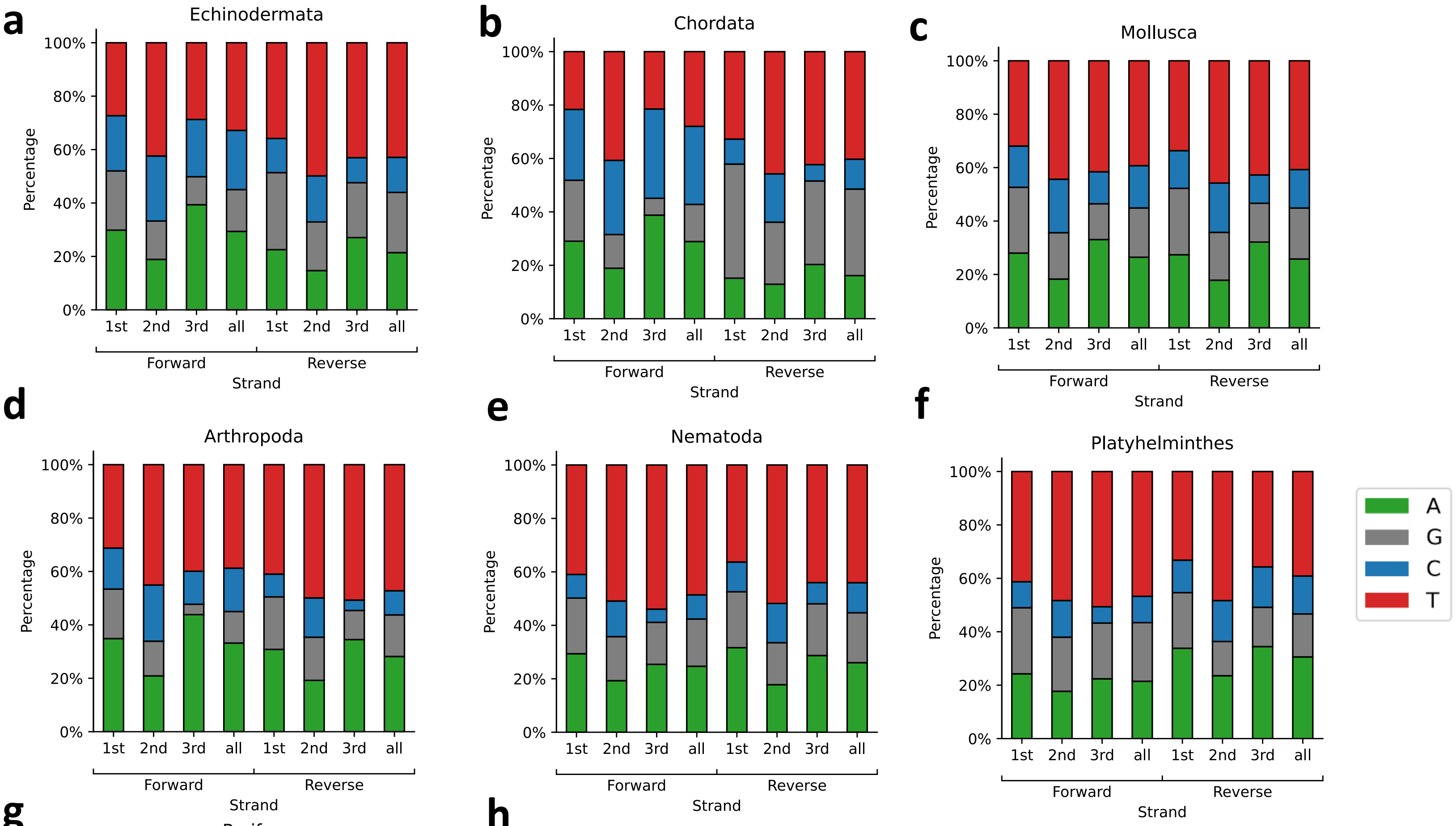

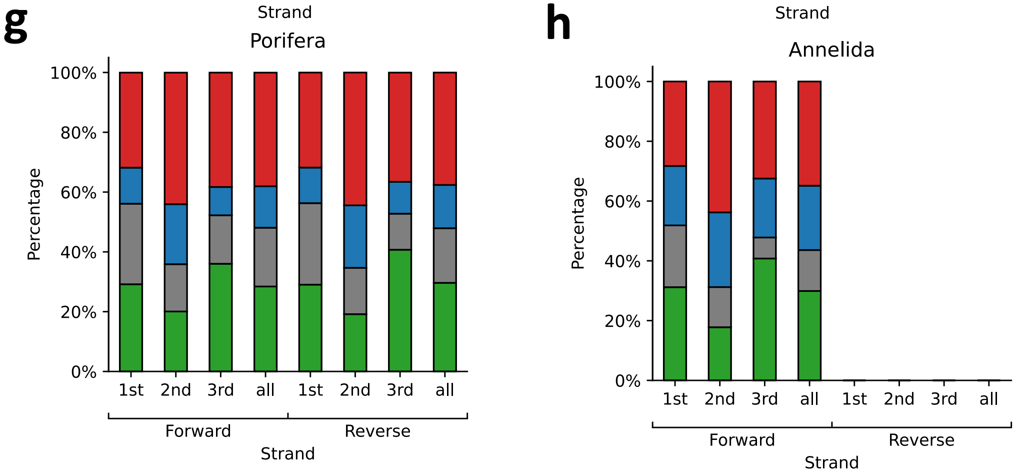

Supplementary Figure S2. Base composition of mtDNA protein coding genes across Metazoa. Stacked bar plots showing the prevalence of each nucleotide per phylum, in every codon position (1 to 3) and in total. The nucleotide frequencies are measured for genes in the forward and reverse mtDNA strands separately. See Supplementary Data S12 for raw data (https://figshare.com/projects/Shtolz_2022_mtDNA_evolutionary_rearrangements/156008)

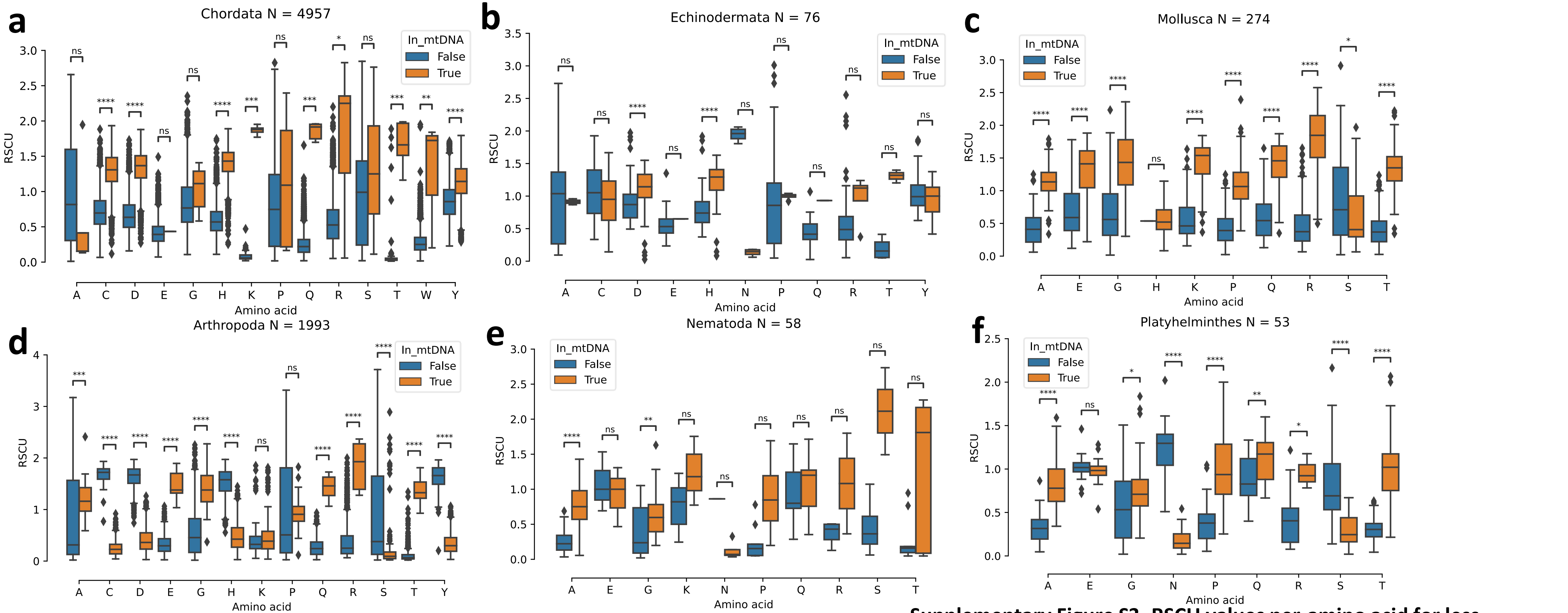

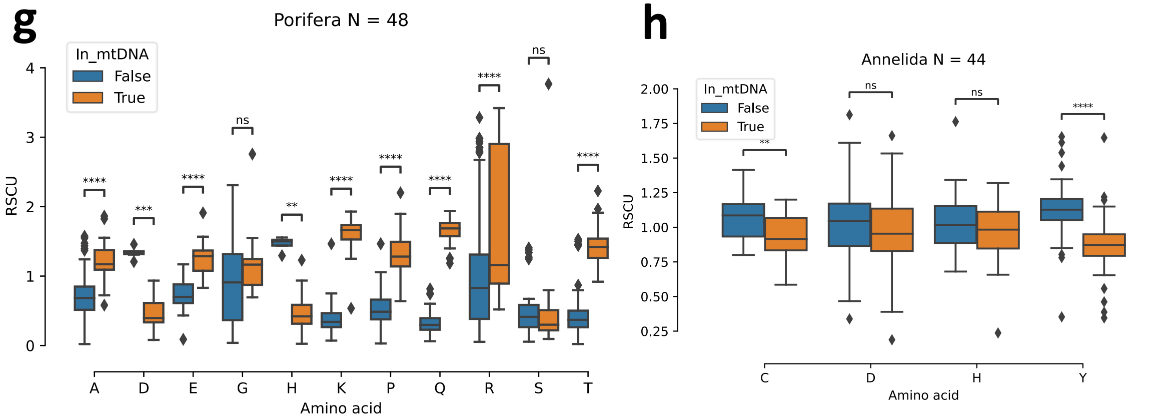

Supplementary Figure S3. RSCU values per-amino acid for less prevalent codons.

Boxplot comparisons of RSCU values between codons recognized (orange) and unrecognized (blue) by the mt-tRNA, per amino acid, and for each informative phylum. The amino acids were filtered for these having at least two different codons, one recognized and one unrecognized by the mt-tRNAs. Species were filtered for these that have at least two mt-tRNA genes which associate with amino acids that can be translated by at least two different codons. To mitigate the effect of base composition biases on mt-tRNA bias, only codons that do not contain the most prevalent base within each codon position, per strand and phylum were selected. The RSCU values were compared using a two-sided Mann-Whitney test, corrected for multiple testing using the Benjamini-Hodgkin method. P-value ranges are indicated by asterisks. *: 0.01 < p <= 0.05, **: 0.001 <= p < 0.01, ***: 0.0001 < p <= 0.001, ****: p <= 0.0001. Each box extends from the 25th to the 75th percentiles, whiskers denote values within 1.5 interquartile range of the percentiles. Dots denote outliers outside of mentioned ranges.

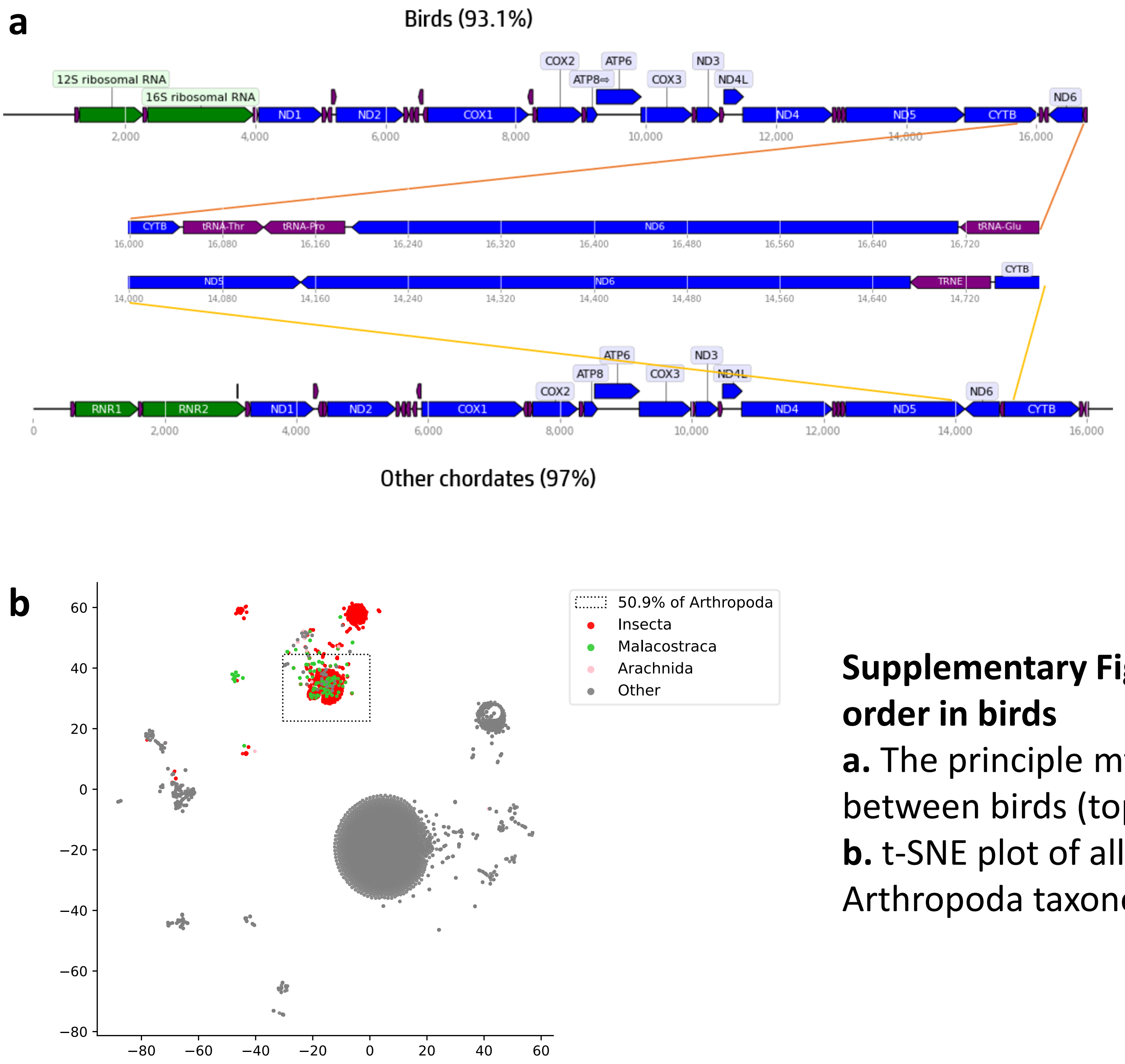

Supplementary Figure S4. Unique mtDNA gene order in birds

a. The principle mtDNA gene order difference between birds (top) and other chordates (bottom).

b. t-SNE plot of all organisms, colored by Arthropoda taxonomic classes.

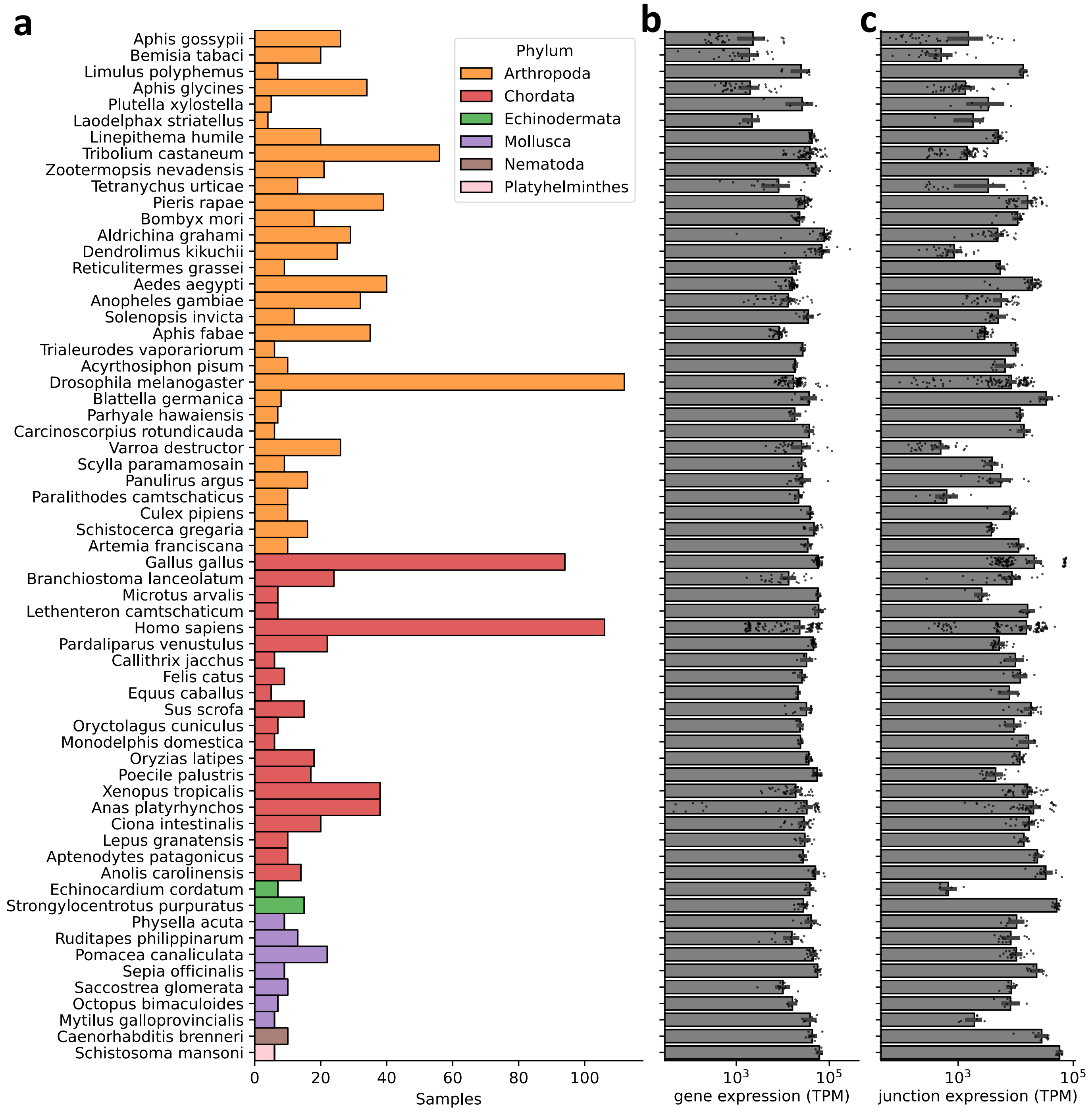

Supplementary Figure S5. Number of RNA-seq samples per organism.
a. The number of RNA-seq samples per organism used in the analysis (post quality control). **b, c.** Bar plot distributions of TPM normalized expression for each organism in logarithmic scale, across all mtDNA genes (**b**) or intergenic junctions (**c**). Dots denote individual samples.

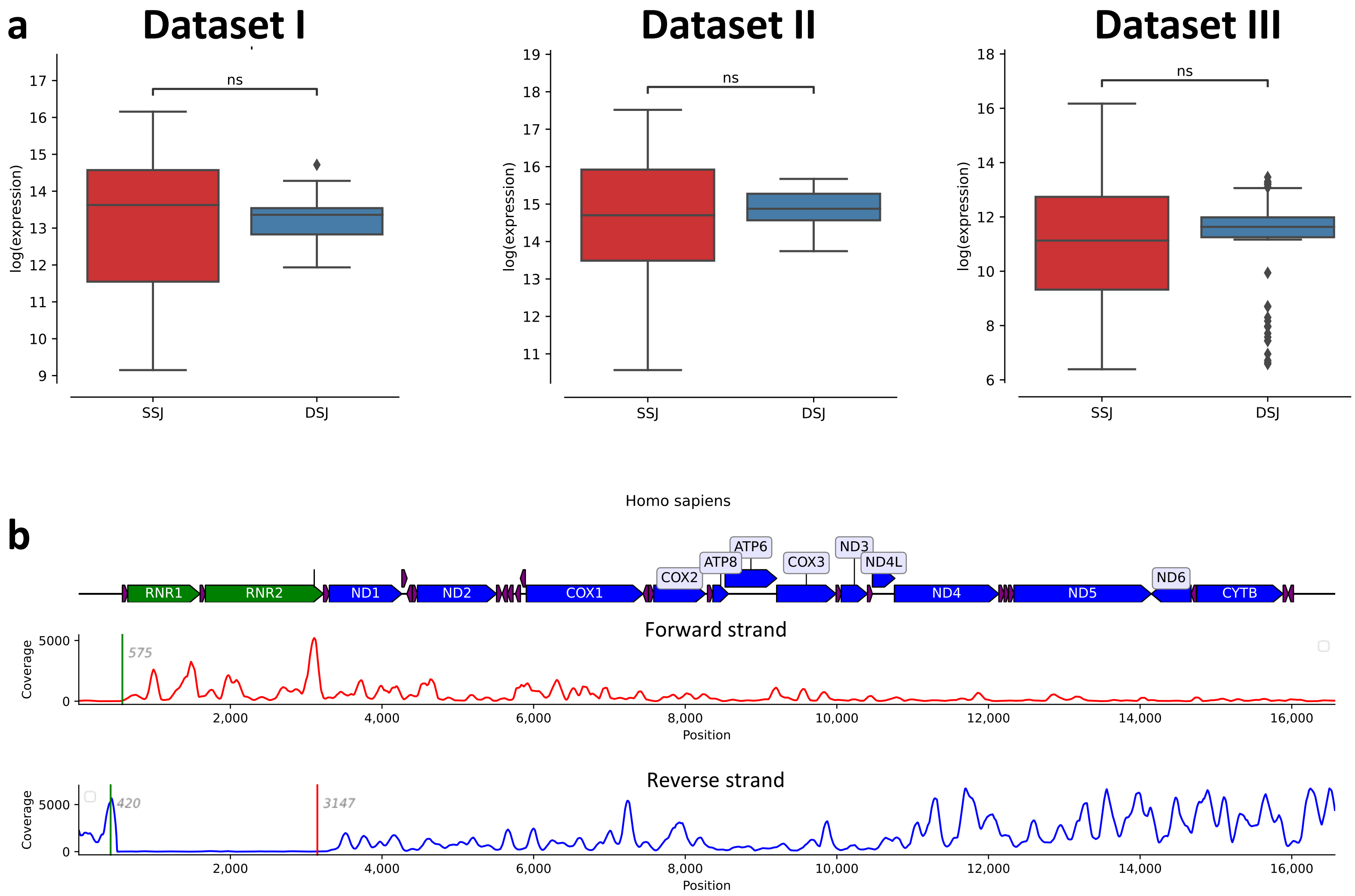

Supplementary Figure S6. Nonsignificant expression difference between SSJ and DSJ in humans.

a. Comparison between DSJ and SSJ expression in three human RNA-seq datasets. **b.** MtDNA coverage of a human PRO-seq sample. The forward strand is in red and the reverse strand in blue. Red and green vertical lines mark predicted termination and initiation sites, respectively. Each box extends from the 25th to the 75th percentiles, whiskers denote values within 1.5 interquartile range of the percentiles. Dots denote outliers outside of mentioned ranges.

Taxon	Mitozoa (2012)	Current database (pre filteration)	% Increase
Acanthocephala	1	10	900
Annelida	18	79	339
Arthropoda	527	2770	426
Brachiopoda	5	5	0
Bryozoa	3	8	167
Cephalochordata	9	9	0
Chaetognatha	5	5	0
Cnidaria	70	213	204
Ctenophora	1	4	300
Echinodermata	29	80	176
Echiura	2	2	0
Entoprocta	2	4	100
Hemichordata	3	5	67
Hyperotreti	2	11	450
Mollusca	148	491	232
Myzostomida	2	2	0
Nematoda	75	88	17
Nemertea	5	13	160
Onychophora	6	6	0
Platyhelminthes	45	66	47
Porifera	31	56	81
Priapulida	2	2	0
Rotifera	3	3	0
Sipuncula	3	3	0
Tardigrada	2	2	0
Tunicata	13	13	0
Vertebrata	2062	5233	154
Xenoturbellida	1	11	1000

Supplementary Table S1. Number of species per taxa in our database in comparison to MitoZoa

The table shows a comparison of the number of species within 28 selected species, in the MitoZoa database (last updated in 2012), as compared to the database used throughout this article. The third column shows the % increase in the number of species.
