# Supplementary material for: The metazoan landscape of mitochondrial DNA gene order and content is shaped by selection and affects mitochondrial transcription
Source: Commun Biol. 2023 Jan 23;6:93. doi: 10.1038/s42003-023-04471-4 (PMC9871016; doi:10.1038/s42003-023-04471-4)
Supplement: Supplementary file 3 — Description of Additional Supplementary Files [file 42003_2023_4471_MOESM3_ESM.pdf]

Description of Additional Supplementary Files

File name: Supplementary Data S1
Description: All newly analyzed species within the database used.

File name: Supplementary Data S2
Description: All next generation sequencing experiments analyzed.
