# Supplementary material for: The metazoan landscape of mitochondrial DNA gene order and content is shaped by selection and affects mitochondrial transcription
Source: Commun Biol. 2023 Jan 23;6:93. doi: 10.1038/s42003-023-04471-4 (PMC9871016; doi:10.1038/s42003-023-04471-4)
Supplement: Supplementary file 6 — Reporting Summary [file 42003_2023_4471_MOESM6_ESM.pdf]

nature portfolio

Corresponding author(s): Dan Mishmar, PhD

Last updated by author(s): Dec 25, 2022

Reporting Summary

Nature Portfolio wishes to improve the reproducibility of the work that we publish. This form provides structure for consistency and transparency in reporting. For further information on Nature Portfolio policies, see our Editorial Policies and the Editorial Policy Checklist.

Statistics

For all statistical analyses, confirm that the following items are present in the figure legend, table legend, main text, or Methods section.

n/a	Confirmed	
☒	☐	The exact sample size (*n*) for each experimental group/condition, given as a discrete number and unit of measurement
☒	☐	A statement on whether measurements were taken from distinct samples or whether the same sample was measured repeatedly
☐	☒	The statistical test(s) used AND whether they are one- or two-sided<br>*Only common tests should be described solely by name; describe more complex techniques in the Methods section.*
☐	☒	A description of all covariates tested
☐	☒	A description of any assumptions or corrections, such as tests of normality and adjustment for multiple comparisons
☐	☒	A full description of the statistical parameters including central tendency (e.g. means) or other basic estimates (e.g. regression coefficient) AND variation (e.g. standard deviation) or associated estimates of uncertainty (e.g. confidence intervals)
☐	☒	For null hypothesis testing, the test statistic (e.g. *F*, *t*, *r*) with confidence intervals, effect sizes, degrees of freedom and *P* value noted<br>*Give P values as exact values whenever suitable.*
☒	☐	For Bayesian analysis, information on the choice of priors and Markov chain Monte Carlo settings
☒	☐	For hierarchical and complex designs, identification of the appropriate level for tests and full reporting of outcomes
☐	☒	Estimates of effect sizes (e.g. Cohen's *d*, Pearson's *r*), indicating how they were calculated

Our web collection on statistics for biologists contains articles on many of the points above.

Software and code

Policy information about availability of computer code

Data collection	N/A
Data analysis	All sequence data analyzed in the study were downloaded from both the NCBI organelle database (https://www.ncbi.nlm.nih.gov/genome/organelle/) and the Mitozoa website (http://srv00.recas.ba.infn.it/mitozoa/). RNA-seq experimental data were downloaded according to accession numbers listed in Supplementary Table S3. tRNA validation was applied to all identified tRNA genes using two different algorithms, tRNAscan-SE (v2.0.5) and ARWEN (v1.2.3). To study the evolutionary landscape of mtDNA gene organization changes during the evolution of Metazoa, a pairwise distance matrix was created using the common interval rearrangement explorer, CREx 46 (v1.0.0). To visualize the N x N distance matrix generated, a Python implementation of the t-SNE algorithm was used, which is part of the scikit-learn (v0.23.1) with a chosen perplexity value of 30, chosen because it yielded the most informative visualization.To detect motifs that are over-represented in arthropods that have alternating gene block organization, we used XSTREME (v5.4.1), which combines motif discovery with motif enrichment analysis and clustering. All code written for the sake of analyses in the current manuscript was deposited in GitHub: https://github.com/Noam-St/2022_Metazoan_mtDNA_Project

For manuscripts utilizing custom algorithms or software that are central to the research but not yet described in published literature, software must be made available to editors and reviewers. We strongly encourage code deposition in a community repository (e.g. GitHub). See the Nature Portfolio guidelines for submitting code & software for further information.

Data

Policy information about availability of data

All manuscripts must include a data availability statement. This statement should provide the following information, where applicable:

- Accession codes, unique identifiers, or web links for publicly available datasets
- A description of any restrictions on data availability
- For clinical datasets or third party data, please ensure that the statement adheres to our policy

All sequence data analyzed in the study were downloaded from both the NCBI organelle database (https://www.ncbi.nlm.nih.gov/genome/organelle/) and the Mitozoa website (http://srv00.recas.ba.infn.it/mitozoa/). RNA-seq experimental data were downloaded according to accession numbers listed in Supplementary Table S3.

Field-specific reporting

Please select the one below that is the best fit for your research. If you are not sure, read the appropriate sections before making your selection.

☒ Life sciences ☐ Behavioural & social sciences ☐ Ecological, evolutionary & environmental sciences

For a reference copy of the document with all sections, see nature.com/documents/nr-reporting-summary-flat.pdf

Life sciences study design

All studies must disclose on these points even when the disclosure is negative.

Sample size	To construct a comprehensive database of metazoan mitochondrial gene features (i.e. annotated genes, their described mtDNA genes order) of as many organisms as possible, all available mtDNA features were extracted and combined from both the NCBI Organelle database and the MitoZoa database (v2.0.0), yielding the complete mtDNA features of 9657 different metazoan organisms. Since organisms with at least a single unvalidated tRNA, incomplete or fragmented mtDNA, or CDS-containing introns were excluded from further analysis, we retained 8053 different metazoan organisms for subsequent analyses. Complete description of the data analysis is available in the "Creation of a database of mitochondrial DNA features in metazoans" subsection, within the Methods chapter.
Data exclusions	Since organisms with at least a single unvalidated tRNA, incomplete or fragmented mtDNA, or CDS-containing introns were excluded from further analysis, we retained 8053 different metazoan organisms for subsequent analyses. Complete description of the data analysis is available in the "Creation of a database of mitochondrial DNA features in metazoans" subsection, within the Methods chapter.
Replication	N/A
Randomization	To generate distributions of expected and observed mitochondrial genome architecture (AR) change rates, while considering large variation in sample sizes of the available metazoan classes (ranging from 21 in Ophiuroidea to 2471 organisms in Actinopterygii), we randomly sampled 21 organisms from each class 10,000 times using a custom R script and calculated AR change rate for each class with and without prior shuffling for the expected and observed populations respectively. Complete description of the data analysis is available in the "Calculation of mitochondrial genome architecture change rates and permutation tests" subsection, within the Methods chapter.
Blinding	N/A

Reporting for specific materials, systems and methods

We require information from authors about some types of materials, experimental systems and methods used in many studies. Here, indicate whether each material, system or method listed is relevant to your study. If you are not sure if a list item applies to your research, read the appropriate section before selecting a response.

Materials & experimental systems

n/a	Involved in the study
☒	☐ Antibodies
☒	☐ Eukaryotic cell lines
☒	☐ Palaeontology and archaeology
☒	☐ Animals and other organisms
☒	☐ Human research participants
☒	☐ Clinical data
☒	☐ Dual use research of concern

Methods

n/a	Involved in the study
☒	☐ ChIP-seq
☒	☐ Flow cytometry
☒	☐ MRI-based neuroimaging
